# Effect of Neem Leaf Paste Application on Dandruff

**DOI:** 10.7759/cureus.80685

**Published:** 2025-03-16

**Authors:** Pravin B Dani, Vishal K Ghorpade

**Affiliations:** 1 Department of Medical Surgical Nursing, Bharati Vidyapeeth (Deemed to be University) College of Nursing, Sangli, IND

**Keywords:** application, dandruff, effect, female students, neem leaves paste

## Abstract

Background: Dandruff is a widespread scalp issue that affects many people worldwide, often causing itching and discomfort. It is linked to the presence of certain microorganisms on the scalp, especially *Malassezia* yeast, which interacts with skin cells to trigger dandruff. This condition can lead patients to experience health concerns and social or emotional distress. Common treatments include antifungal shampoos. Neem leaves have been selected for their natural ability to fight germs, reduce inflammation, and support immune health. They contain beneficial compounds that help treat infections, aid healing, and have long been used in traditional medicine as a natural remedy. This study aims to evaluate the effectiveness of neem leaf paste in treating scalp dandruff among female students.

Materials and methods: A quasi-experimental pre-test post-test control group study was conducted on female students aged 18-24 with minimal, moderate, and severe dandruff levels. The modified Van Abbe’s scale was used to assess the dandruff level. A total of 100 female students were selected using non-probability purposive sampling with no history of allergy to neem leaf formulations. The primary objective was to reduce the dandruff score. The dandruff levels were assessed before and after the application of neem leaf paste at baseline, Week 1, and Week 2. Statistical analysis was performed using frequency and percentage for demographic variables, and a t-test was used to compare dandruff levels between the control and experimental groups. A p-value of 0.05 was considered statistically significant.

Results: The study included 50 female students in the experimental group and 50 in the control group. Before applying neem leaf paste, 66% (n=33) in the experimental group and 60% (n=30) in the control group had a moderate level of dandruff, while 10% (n=5) and 14% (n=7) in the respective groups had severe dandruff. After the intervention, 44% (n=22) in the experimental group had no dandruff, compared to just 4% (n=2) in the control group. Additionally, 42% (n=21) in the experimental group had a minimal level of dandruff, whereas 28% (14 participants) in the control group had the same. Only 14% (n=7) in the experimental group still had moderate dandruff, compared to 60% (n=30) in the control group. The control group also had 8% (n=4) with severe dandruff, while none remained at this level in the experimental group. After one week, the mean dandruff score significantly dropped to 0.7 in the experimental group, compared to 1.72 in the control group (p-value = 0.00001), indicating a statistically significant reduction in dandruff severity due to neem leaf paste application.

Conclusion: The application of neem leaf paste was effective in managing dandruff among the participants. Neem leaf paste, known for its antimicrobial and anti-inflammatory properties, offers a cost-effective and natural alternative for managing dandruff. Its effectiveness in reducing dandruff levels among study participants highlights its potential as a safe and accessible treatment option.

## Introduction

Dandruff is a common scalp problem that affects many people, especially after puberty. While it is not a serious medical condition, it can cause white flakes, itching, and discomfort, which may lower a person’s confidence. Understanding its causes is important for effective treatment and management. Dandruff can be caused by both internal and external factors. Internal factors include hormonal changes that affect oil production on the scalp, making it easier for dandruff to develop. Certain food allergies and environmental triggers can make the scalp more sensitive, while a diet high in fried foods, sugary drinks, and dairy products may lead to poor scalp health. Stress, anxiety, and lack of sleep can weaken the immune system, increasing the chances of dandruff. External factors also play a big role. Using too many hair products like sprays, gels, and mousse can irritate the scalp. Poor hair care, such as not washing hair regularly or not rinsing properly, can lead to oil and dead skin buildup. Weather conditions also affect dandruff cold weather and indoor heating can dry out the scalp, while humid weather can encourage fungal growth. Additionally, using alcohol-based hair lotions, heated styling tools, or wearing tight hats and scarves can further irritate the scalp and contribute to dandruff. Understanding and managing these factors can help in keeping the scalp healthy and dandruff-free [1].

The yeast-like fungus *Malassezia* (formerly *Pityrosporum ovale*) naturally resides on the scalp but can proliferate excessively under conditions like increased sebum production or a weakened immune system, leading to scalp irritation, inflammation, itching, and flaking-symptoms commonly associated with dandruff and severe cases like seborrheic dermatitis. The study titled "Neem: A Tree for Solving Global Problems" by the National Research Council highlights neem's vast medicinal potential, including its antifungal properties, which may help combat *Malassezia*-induced scalp conditions. Recognized for its role in sustainable agriculture, traditional and modern medicine, and environmental conservation, neem presents a promising natural remedy for dandruff while also contributing to broader health and ecological benefits. The study underscores the need for further research to harness neem’s full potential in addressing global challenges, including healthcare solutions like scalp and skin treatments [2].

The study by Nawaf et al. on the prevalence of dandruff among the pupils and staff of selected public schools in Katsina State provides valuable epidemiological insights into dandruff occurrence, identifying *Malassezia* species,* Staphylococcus aureus, *and *Propionibacterium *as contributing microbial factors. The study found a higher prevalence among males (51%) than females (49%) and a significant association with socioeconomic conditions, emphasizing the need for improved hygiene and sterilization practices to prevent its spread [3].

A study by Sinaga and Liana explored the relationship between stress and dandruff among students but found no significant correlation. While stress may not directly cause dandruff, both studies highlight contributing factors such as environmental conditions and microbial activity. These findings reinforce the need for holistic dandruff prevention strategies, including scalp hygiene, antimicrobial treatments, and awareness campaigns to mitigate risk factors like socioeconomic status and personal care habits [4].

Ahmad et al. (2019) highlight the extensive medicinal properties of the neem tree (*Azadirachta indica*), often referred to as the village pharmacy of South Asia. Growing in tropical and subtropical regions, neem has been used in Ayurvedic, Siddha, and herbal medicine for thousands of years. The study emphasizes neem’s therapeutic potential, citing its antiviral, antifungal, antibacterial, anti-inflammatory, and anti-dermatitic properties. With approximately 135 bioactive compounds identified across various parts of the tree, neem formulations effectively treat numerous conditions, including ulcers, eczema, sores, burns, and infections, underscoring their significance as a versatile and holistic natural remedy [5].

This study highlights neem’s potential as a natural and sustainable remedy for dandruff, focusing on its practical use and environmental benefits. By examining neem’s antidandruff properties, the research aims to offer a safe and holistic solution that improves scalp health, minimizes the use of chemicals, and enhances overall well-being.

## Materials and methods

A quasi-experimental study with a pre-test and post-test control group design was conducted to evaluate the effectiveness of neem leaf paste in reducing dandruff severity among female students aged 18 to 24 years with mild, moderate, to severe dandruff. Participants were selected using a non-probability purposive sampling technique, resulting in a total sample size of 100. The sample size was determined based on feasibility, available resources, and the need for statistical power to detect a meaningful difference between groups. Inclusion criteria required participants to have moderate to severe dandruff, as assessed by the modified Van Abbe's scale, and no known allergies to neem leaf formulations. To eliminate confounding factors, individuals undergoing any form of hair treatment were excluded from the study.

The study was approved by the institutional ethics committee of Bharati Vidyapeeth (Deemed to be University) College of Nursing, Sangli, under approval number EC/NEW/INST/2024/MH/0414. Before participation, all individuals were provided with detailed information about the study’s purpose, procedures, potential risks, and benefits. They were informed of their right to withdraw from the study at any time without penalty. After receiving this information, participants voluntarily signed an informed consent form, indicating their understanding and agreement to participate. This ethical procedure ensured that the rights and well-being of the participants were respected throughout the study.

To apply neem leaf paste, fresh neem leaves were washed and ground into a smooth paste with water. The paste was then evenly applied to the scalp, covering all dandruff-affected areas. Participants in the experimental group were instructed to moisten their scalps before applying 15 millilitres (about three tablespoons) of freshly prepared neem leaf paste. They were guided to massage it thoroughly into their scalp, leave it on for 15 minutes, and then rinse it off with water. This process was repeated three times a week for two weeks under supervision to ensure protocol adherence. Participants in the control group received no intervention during the study. Dandruff severity was evaluated at three time points using the modified Van Abbe’s scale: at baseline (pre-test), after one week of treatment (Day 7), and after two weeks of treatment (Day 15). This approach ensured consistent and objective measurements of dandruff severity for all participants.

The collected data were analyzed using both descriptive and inferential statistical methods. Descriptive statistics, including frequency and percentage, were used to summarize the demographic characteristics of the participants. To compare the mean dandruff scores between the experimental and control groups at each time point, an independent t-test was conducted. This test assessed the differences in dandruff severity between the two groups before and after the intervention. A p-value of less than 0.05 was considered statistically significant, ensuring the reliability and validity of the findings and confirming that the results were not due to chance.

## Results

The data was analyzed based on the study's objectives, which included comparing dandruff levels between the experimental and control groups, assessing dandruff severity in the experimental group before and after applying neem leaf paste, and identifying the frequency and percentage distribution of demographic characteristics.

There were 100 participants in total, with 50 in the experimental group and 50 in the control group. In the experimental group, 27 participants (54%) were aged between 18 and 22, while 23 participants (46%) were aged between 22 and 24. In the control group, 25 participants (50%) were evenly distributed between the age groups of 18-22 and 22-24. None of the participants in either group reported any allergies to neem leaves. In the experimental group, 10 participants (20%) experienced dandruff continuously, while 40 participants (80%) had intermittent dandruff. Similarly, in the control group, 7 participants (14%) had continuous dandruff, and 43 participants (86%) had intermittent dandruff. The demographic details are presented in Table 1.

**Table 1:** Frequency and percentage distribution of demographical variables in experimental and control group.

Sr. No.	Demographic variables		Experimental group		Control group	
			f	%	f	%
1.	Age (in years)	18 - 22	27	54%	25	50%
		22 - 24	23	46%	25	50%
2.	Any history of allergy with neem leaves	Yes	0	0%	0	0%
		No	50	100%	50	100 %
3.	Frequency of dandruff	Continuous	10	20%	7	14%
		Intermittent	40	80%	43	86%

The assessment of dandruff before the application of neem leaf paste was done for both the experimental and control groups. The following results were observed among female students on Day 1. In the experimental group, 12 students (24%) had a minimal level of dandruff, while 13 students (26%) in the control group had the same. Most of the students, 33 (66%) in the experimental group and 30 (60%) in the control group, had a moderate level of dandruff. A small number of students, 5 (10%) in the experimental group and 7 (14%) in the control group, had the highest level of dandruff. In both groups, most of the girls had a moderate level of dandruff before the application of the neem leaf paste. The detailed assessment of dandruff among female students in both groups before the application of neem leaves is shown in Table 2.

**Table 2:** Assessment of the level of dandruff among female Students in the experimental and control groups before intervention

	Pre-test	Experimental Group		Control Group	
		f	%	f	%
Level of dandruff	0 (No dandruff)	0	0%	0	0%
	1 (Minimum level of dandruff)	12	24%	13	26%
	2 (Moderate level of dandruff)	33	66%	30	60%
	3 (Highest level of dandruff)	5	10%	7	14%

By observing the level of dandruff among female students after the application of neem leaf extract, the following results were obtained: On Day 7, 22 students (44%) in the experimental group and 2 students (4%) in the control group had no dandruff. Most of the students, 21 (42%) in the experimental group and 14 (28%) in the control group had a minimal level of dandruff. A small number of students, 7 (14%) in the experimental group and 30 (60%) in the control group had a moderate level of dandruff. Additionally, in the control group, 4 students (8%) had the highest level of dandruff after the intervention. Most of the students in the experimental group experienced no dandruff or minimal dandruff after the application of neem leaf paste. Therefore, it can be concluded that the level of dandruff decreased among female students in the experimental group compared to the control group after the neem leaf paste application. The effect of neem leaf paste on dandruff among female students in both the experimental and control groups after the intervention is shown in Table 3.

**Table 3:** Assessment of the Level of Dandruff Among Female Students in the Experimental and Control Groups After the Intervention

	Post-test	Experimental group		Control group	
		f	%	f	%
Level of dandruff	0 (No dandruff)	22	44%	2	4%
	1 (Minimum level of dandruff)	21	42%	14	28%
	2 (Moderate level of dandruff)	7	14%	30	60%
	3 (Highest level of dandruff)	0	0%	4	8%

By Day 15, after applying neem leaf paste, the experimental group showed a mean dandruff score of 0.7 with a standard deviation (SD) of 0.7071. In the control group, the mean dandruff score was 1.72 with an SD of 0.6712. The t-value was 7.3974, and the p-value was 0.00001, which is statistically significant at the 5% level of significance. This indicates a significant difference between the post-test dandruff scores of the experimental and control groups on Day 15. The results clearly show that after applying neem leaf paste, the experimental group experienced a significant improvement in dandruff compared to the control group. The findings reveal that applying neem leaf paste was effective in reducing dandruff among female students. Out of the 100 participants (50 in the experimental group and 50 in the control group), most of the students experienced intermittent dandruff. The comparison of post-test dandruff scores between the experimental and control groups is shown in Table 4.

**Table 4:** Comparison of post-test score of effect of neem leaves paste on dandruff in experimental and control group among female students.

Post-test	Mean	SD	t-value	p-value	Result
Experimental group	0.7	0.7071	7.3974	0.00001	Significant
Control group	1.72	0.6712			

## Discussion

The findings of this study clearly demonstrate the effectiveness of neem leaf paste in reducing dandruff levels among female students. Before the intervention, most participants in both the experimental and control groups exhibited moderate levels of dandruff (66% in the experimental group and 60% in the control group). A smaller proportion had minimum dandruff levels (24% in the experimental group and 26% in the control group), while the least number of participants had the highest levels of dandruff (10% in the experimental group and 14% in the control group). This baseline distribution emphasizes that moderate dandruff was the predominant condition before applying neem leaf paste.

After the intervention (Day 15), a significant reduction in dandruff levels was observed in the experimental group compared to the control group. The experimental group showed a remarkable improvement, with 44% of participants having no dandruff and 42% exhibiting only a minimum level of dandruff. In contrast, only 4% of the control group participants experienced no dandruff, and 28% had minimum dandruff levels. Notably, 60% of the control group participants still had moderate dandruff, and 8% retained the highest dandruff level, highlighting the lack of significant improvement in the absence of neem leaf paste application.

Asghar et al. conducted a study highlighting neem as an ancient medicinal plant with wide-ranging pharmacological and therapeutic properties. Various parts of neem, such as leaves, fruit, bark, seeds, and flowers, contain biologically active compounds that effectively prevent and treat numerous health disorders. Neem offers a range of benefits, including anti-cancer, anti-inflammatory, anti-malarial, antibacterial, antiviral, antifungal, antioxidant, hepatoprotective, and neuropharmacological effects. It is generally well-tolerated, with rare allergic reactions reported, and its stomach-friendly nature makes it suitable for prolonged use. Neem’s broad therapeutic potential positions it as a valuable, natural remedy for addressing various health concerns [6].

The results of the study align with existing literature highlighting neem’s antifungal, antimicrobial, and anti-inflammatory properties, which are effective in targeting *Malassezia* yeast, a key contributor to dandruff. These findings suggest that neem leaf paste can be an effective natural remedy for managing dandruff, supporting its potential as an alternative to chemical-based treatments. Additionally, neem leaf paste offers a cost-effective and accessible solution, making it a sustainable approach for individuals seeking a natural treatment for dandruff. This study reinforces the value of neem in promoting scalp health and providing an environmentally friendly alternative to conventional treatments [7].

A similar study conducted by Godse et al. investigated the effectiveness of a 2.5% selenium sulfide shampoo as a topical antifungal formulation for managing dandruff. The study found that the shampoo was effective in reducing dandruff and alleviating associated symptoms such as itching, oiliness, and greasiness. Additionally, the formulation demonstrated a good safety profile among Indian participants with dandruff, making it a reliable treatment option for managing dandruff-related scalp conditions. This study underscores the therapeutic potential of selenium sulfide in improving scalp health and minimizing dandruff symptoms in a specific population [8].

Similarly, in a laboratory study conducted by Niharika et al., the antifungal properties of neem leaf extract were explored. The study demonstrated that neem leaf extract effectively inhibits the growth of *Pityrosporum ovale* (*P. ovale*), a fungus primarily responsible for causing dandruff. The research highlighted the potential of neem leaf extract as a natural remedy for dandruff, providing evidence of its effectiveness in targeting the underlying fungal infection that contributes to dandruff development. This finding supports the use of neem as a therapeutic agent for managing dandruff and promoting scalp health [9].

A study conducted by Kumara et al. explores dandruff, a scalp disorder characterized by itching and an abnormal, rapid turnover of the outermost skin layer. The condition is primarily caused by* Malassezia* fungi. Various herbal remedies have traditionally been used for treatment, with studies evaluating their effectiveness against dandruff-causing fungi. This review summarizes research on medicinal plants in Sri Lanka with anti-dandruff properties, including *Punica granatum*, *Mentha piperita*, *Bacopa monnieri*, *Asparagus racemosus*, *Azadirachta indica,* and others. While in vitro studies show promising antifungal activity, clinical trials remain limited. However, evidence suggests that combined plant extract formulations are more effective than single herb preparations [10].

A study conducted by Moin et al. Explores the medicinal significance of neem (*Azadirachta indica* A. Juss.). Known as a sacred gift of nature, neem has been widely used in traditional Indian medicine for treating various ailments. Every part of the neem tree possesses medicinal properties, with bioactive compounds like nimbin and azadirachtin exhibiting strong antimicrobial, insecticidal, and antifungal effects. Neem is beneficial for heart diseases, hepatitis, malaria, psoriasis, and ulcers. This study reviews neem’s pharmacological activities, clinical applications, and its potential role in medicine [11].

A study conducted by Nirmala Nithya et al. Explores dandruff, a common scalp condition caused by *Malassezia furfur*, sebaceous secretion, and individual sensitivity. Dandruff flakes were isolated from patients and cultured on potato dextrose agar and Sabouraud’s dextrose agar. The antidandruff activity of *Piper cubeba*,* Cissus quadrangularis*, and *Bauhinia vahlii *was evaluated using well diffusion and broth dilution assays. *Piper cubeba* methanol extract exhibited significant activity, with a minimum inhibitory concentration of 100 µg/ml and IC50 of 800 µg/ml. Partial purification via TLC revealed an active compound with an Rf value of 0.705 [12].

The statistical analysis supports these observations. The mean dandruff score for the experimental group (0.7 ± 0.7071) was significantly lower than that of the control group (1.72 ± 0.6712), with a t-value of 7.3974 and a p-value of 0.00001. These results indicate a statistically significant reduction in dandruff levels in the experimental group, confirming the effectiveness of neem leaf paste application as a treatment.

The study was limited to female students within a specific age range and geographic location, which may affect the generalizability of the findings. Additionally, the control group did not receive a placebo intervention, which could introduce biases related to the placebo effect. Furthermore, the study did not account for potential confounders such as diet, stress levels, scalp type, and hair care routines, and the absence of multivariate analysis limits the robustness of the findings. The relatively small sample size and short follow-up period may also impact the reliability of the results, while the subjective assessment of dandruff severity could introduce measurement bias.

## Conclusions

This study confirms that neem leaf paste is an effective natural remedy for reducing dandruff levels among female students. A significant improvement was observed in the experimental group, where most participants experienced no or minimum dandruff after the intervention. The study highlights several advantages of neem leaf paste as a treatment for dandruff, including its non-pharmacological nature, cost-effectiveness, and eco-friendly properties. These findings emphasize the potential of neem as a safe, accessible, and sustainable solution for dandruff management. Moreover, the positive results underscore the importance of incorporating natural remedies in dermatological care to improve health outcomes and reduce reliance on chemical treatments. Future research could further explore the long-term effects of neem leaf paste, its mechanisms of action, and its combination with other natural remedies for more comprehensive dandruff management. Neem leaf paste is an effective treatment for dandruff, making it a valuable addition to the available solutions for this common scalp condition.
